# Sex-based disparities in the association between uric acid levels and anxiety: a cross-sectional analysis of nationwide data in Korea

**DOI:** 10.1186/s12888-025-06474-3

**Published:** 2025-01-23

**Authors:** Soohyun Park, Joo O. Kim, Gyu Nam Park, Jae Won Oh, San Lee

**Affiliations:** 1 Yonsei University College of Medicine, Seoul, Republic of Korea; 2 Department of Psychiatry and Institute of Behavioral Science in Medicine, Yonsei University College of Medicine, Seoul, Republic of Korea; 3 Republic of Korea Navy, Gyeryong, Republic of Korea; 4 Department of Psychology, University of Utah Asia Campus, Incheon, Republic of Korea; 5 Department of Psychiatry, Yonsei University College of Medicine, 50-1 Yonsei-ro, Seodaemun-gu, Seoul, 03722 Republic of Korea

**Keywords:** Uric acid, Anxiety disorder, Antioxidant, Sex, KNHANES

## Abstract

**Background:**

Uric acid has antioxidant properties, and several studies have suggested its neuroprotective effects. Despite reports of increased oxidative damage and decreased antioxidants in anxiety disorders, findings remain inconclusive. This study investigated the association between serum uric acid levels and anxiety symptoms, stratified by sex, using nationwide data from South Korea.

**Methods:**

Data were derived from the Korea National Health and Nutrition Examination Survey, which included 2,228 males and 2,805 females. Presence of anxiety symptoms was defined as a Generalized Anxiety Disorder 7-item scale (GAD-7) score of ≥ 10. Study participants were categorized into three groups based on serum uric acid levels: 1 (lowest) to 3 (highest). Multivariable logistic regression analyzed the association between uric acid levels and anxiety symptoms, stratified by sex.

**Results:**

Compared to reference group 2, females in group 1 had increased anxiety symptoms (odds ratio (OR) 2.28, 95% confidence interval (CI) 1.48–3.49). When anxiety symptoms were defined as a GAD-7 score of ≥ 5, females in groups 1 (OR 1.68, 95% CI 1.30–2.16) and 3 (OR 1.35, 95% CI 1.04–1.74) both showed more anxiety symptoms than group 2, with a U-shaped relationship between uric acid levels and anxiety symptoms. In males, uric acid levels weren't significantly linked to anxiety symptoms.

**Conclusions:**

This study indicates that low serum uric acid levels are associated with a higher prevalence of anxiety symptoms only in females, suggesting involvement of oxidative stress in anxiety disorders and its sex-based variation.

**Supplementary Information:**

The online version contains supplementary material available at 10.1186/s12888-025-06474-3.

## Background

Anxiety disorders are common mental disorders with a 12-month prevalence of approximately 18% [1] and are the most prevalent psychiatric disorders [2]. Conditions such as generalized anxiety disorder (GAD), panic disorder, specific phobias, and social anxiety disorder affect millions of people globally. Anxiety disorders are highly comorbid with one another and with other mental disorders, such as depression [3]. They are frequently more impairing than physical disorders, yet they are often less likely to be diagnosed and treated [4]. The disability-adjusted life years lost (DALY) for panic disorder, a type of anxiety disorder, was higher than the DALY for numerous neurodegenerative diseases such as Parkinson’s disease [2].

Uric acid (UA), a product of purine metabolism, has traditionally been associated with various diseases, particularly gout and kidney stones [5]. Elevated UA levels have been linked to cardiovascular disease and an increased risk of mortality [6]. However, the impact of UA on health is complex, with some evidence suggesting that it also possesses beneficial health properties. Recent research has unveiled its potential anti-oxidative properties, suggesting that UA may play a protective role against oxidative stress [7], which has implications for various conditions, including neurological disorders, such as Parkinson’s disease [8]. UA may play a crucial role as an antioxidant in the central nervous system (CNS) because of its capacity to stabilize another antioxidant, ascorbic acid, which is prevalent in neurons [9], highlighting its potential significance in psychiatric disorders.

The significant mediators of anxiety in the central nervous system are thought to be norepinephrine, serotonin, dopamine, and gamma-aminobutyric acid [10, 11]. The amygdala plays a critical role in controlling fear and anxiety [10]. Patients with anxiety disorders have been found to show a heightened amygdala response to anxiety cues [12]. However, the exact cause of anxiety disorder remains ambiguous. Although the pathophysiology of anxiety disorder is not fully understood, oxidative stress is a recognized factor in the pathogenesis of anxiety, and increased oxidative damage and lower levels of antioxidants have been reported in relation to anxiety disorders [13, 14]. This suggests that UA may play a role as an antioxidant in relation to anxiety disorder.

In prior research, serum UA did not show a consistent pattern of association with major depressive disorder (MDD) or anxiety disorder apart from social phobia, a subtype of anxiety disorder [15]. Two studies found lower UA in depression [16, 17], but one of these studies were conducted with small groups of participants and also revealed high heterogeneity, meaning that the results are inconsistent [16]. Another study has shown that plasma UA levels in subjects with current MDD and/or anxiety disorder(s) were lower than in controls, and UA levels were inversely associated with all symptom severity and duration measures of anxiety disorder [17]. However, participants were only recruited from the general population in the Netherlands, the population of which is 81% Caucasian Dutch, making the results difficult to generalize to other regions or races. This study included sex and age as sociodemographic factors, but it did not show the difference of the effect of UA between sex groups. Establishing a link between uric acid levels and anxiety symptoms in different genders could shed light on sex-specific factors, such as hormonal fluctuations, metabolic differences, and unique stress responses, enabling more tailored approaches to diagnosing and managing anxiety disorders in women.

Therefore, this study aims to investigate the association between serum UA levels and the prevalence of anxiety symptoms in a large, sex-divided sample representative of the South Korean population, utilizing data from the 2021 Korea National Health and Nutrition Examination Survey (KNHANES). Given that the South Korean population is primarily ethnically Korean (97%), the study focuses on understanding this association within a Korean population.

## Methods

### Study population and data

This study was conducted using data from the 2021 KNHANES. The KNHANES, guided by the Korea Centers for Disease Control and Prevention, is a comprehensive national survey aimed at gauging the health and nutritional condition of Koreans [18]. Drawing from a wide-ranging dataset comparable to the United States’ National Health and Nutrition Examination Survey, the KNHANES provides vital statistics derived from the general population of Korea. Its primary goal is to assess the health and nutritional state of South Koreans and supply crucial information for the creation and assessment of health policies and initiatives in the nation. The methodology involves a meticulously designed questionnaire that explores the demographic and socioeconomic facets of the participants, including aspects such as age, education, occupation, income, marital status, smoking and drinking habits, and past and present illnesses. KNHANES is composed of three component surveys: a health interview, health examination and nutrition survey. The health interview and health examination are performed by trained medical staff and interviewers. Dieticians visit the homes of participants for the nutrition survey. The surveys collect detailed information on socioeconomic status, health behaviours, quality of life, healthcare utilization, anthropometric measures, biochemical profiles, etc. The target population of KNHANES comprises non-institutionalized Korean citizens residing in Korea [18]. Details of the survey are accessible to the public and can be downloaded from the official KNHANES website (http://knhanes.kdca.go.kr/).

**Fig. 1:**
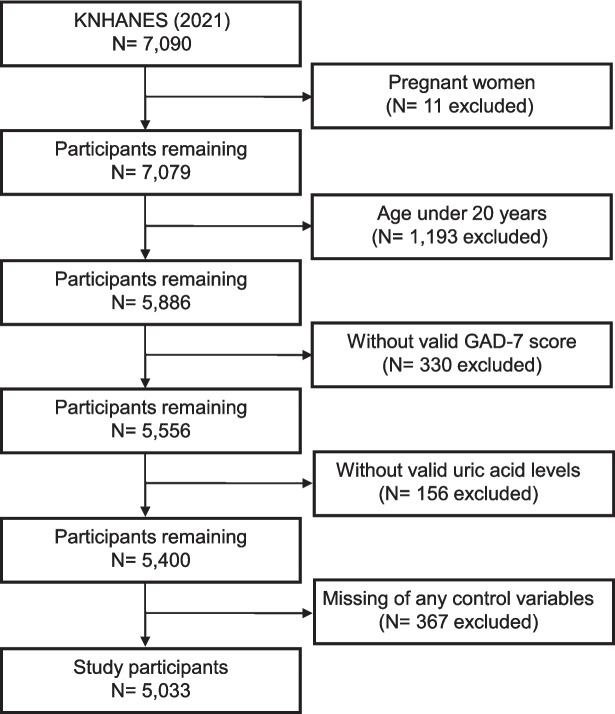
Flow diagram of the study participants. KNHANES, Korea National Health and Nutritional Examination Survey; GAD-7, Generalized Anxiety Disorder- seven-item scale

On an annual basis, the KNHANES is performed across 20 households in each of the 192 regions, aiming to reach around 10,000 individuals who are one year of age or older. The participant pool is categorized into three age-based groups: children, adolescents, and adults. The Generalized Anxiety Disorder seven-item scale (GAD-7), an anxiety disorder screening scale, was introduced in the 2021 KNHANES, and we used the data from that year for this study. In this study, we included 7,090 adult participants and excluded cases of pregnant women (*n* = 11), those under 20 years of age (*n* = 1,193), missing valid GAD-7 score (*n* = 330), missing serum UA data (*n* = 156), and missing any control variables (*n* = 367). The missing control variables were educational attainment (*n* = 281), equalized household income (*n* = 18), BMI (*n* = 68), and we excluded participants with missing control variables in this order. The remaining 5,033 participants (2,228 males, 2,805 females) were eligible for the analysis (Fig. 1).

### Measures

#### Assessment of anxiety symptoms

The presence of anxiety symptoms was assessed using the GAD-7, a seven-item self-administered scale, which has been validated as a reliable anxiety screening tool and a measure of anxiety severity. It is based on the criteria of the Diagnostic and Statistical Manual of Mental Disorders, Fourth Edition [19], which is used to diagnose generalized anxiety disorder (GAD). Each item on the GAD-7 is scored on a scale from 0 to 3, after which the scores are added to yield a total score ranging between 0 and 21. GAD-7 scores of 5, 10, and 15 represent valid thresholds for mild, moderate, and severe GAD, respectively.

In the initial validation study, a score of 10 or higher had a sensitivity of 89% and a specificity of 82% for detecting GAD [20]. GAD is one of the four most common anxiety disorders, together with panic disorder, social anxiety disorder, and posttraumatic stress disorder [21]. Although it was originally developed for GAD, the GAD-7 also proved to have good sensitivity and specificity as a screener for panic, social anxiety, and post-traumatic stress disorders [22]. The area under the curve by receiver operating characteristic (ROC) analysis of the GAD-7 is greatest (0.91) for GAD but also large for panic disorder (0.85), social anxiety disorder (0.83) and PTSD (0.83). GAD-7 was also validated as a reliable anxiety screening tool in Korea [23, 24]. For assessing the four main anxiety disorders, a cutoff score of 10 was used in a previous study, showing decent sensitivity and specificity [25]. Using this cut-off score, individuals with a GAD-7 score of 10 or higher were classified as having anxiety symptoms.

#### Assessment of UA levels

In the 2021 KNHANES, after ≥ 8 h of fasting, blood samples were collected to assess levels of biochemical markers. Venous serum samples were collected in a serum-separating tube, centrifuged at 3000 rpm for 15 min, and stored at 2–8 °C. Serum UA levels were measured within 24 h of sample collection. Serum UA levels were measured by the colorimetric enzymatic (uricase) method using the Labospect 008AS (Hitachi/Japan).

We categorized the study participants (aged ≥ 20 years) into three groups based on tertiles of UA levels. We grouped study participants by sex and separated each sex group into three groups based on UA level. The groups of tertile were made by ranking uric acid levels of the participants and dividing them into three groups as equal in size as possible, making it easy to clarify possible non-linear relationship between UA levels and anxiety symptoms.

### Covariates

Demographic (age), socioeconomic (educational attainment, household income, and marital status), and health-related (body mass index, alcohol consumption status, smoking status, and chronic medical diseases) covariates were included in this study. Medical history was assessed for the presence or absence of hypertension, diabetes mellitus, dyslipidemia, stroke, myocardial infarction, angina, kidney failure, and gout, and these were included as chronic medical diseases.

### Statistical analyses

Statistical analysis was conducted using the SAS software (SAS version 9.4, SAS Institute, Cary, North Carolina, USA). Differences in the general characteristics of the study population were tested using the chi-square test. To examine associations between UA and anxiety, multivariable logistic regression analysis was used. Subgroup analysis was conducted separately by sex and age group (aged below 65/aged 65 or older). We also performed supplementary multivariable logistic regression analysis and subgroup analysis, employing different cutoff scores for the GAD-7, including a threshold of 5. The odds ratio (OR) and 95% confidence interval (CI) were computed, and a *p*-value of < 0.05 was considered significant. The “metafor” package in R (version 4.3.1) was used to visualize data into forest plots. To verify that the use of multivariable logistic regression was appropriate in this study, we conducted the following steps: we assessed multicollinearity among the independent variables by calculating the Variance Inflation Factor (VIF), examined Cook’s Distance and Difference in Betas (DFBETA) to identify any potential outliers or influential points that might disproportionately affect the model, and performed the Hosmer–Lemeshow test to assess the model’s goodness-of-fit.

## Results

### General characteristics

**Fig. 2:**
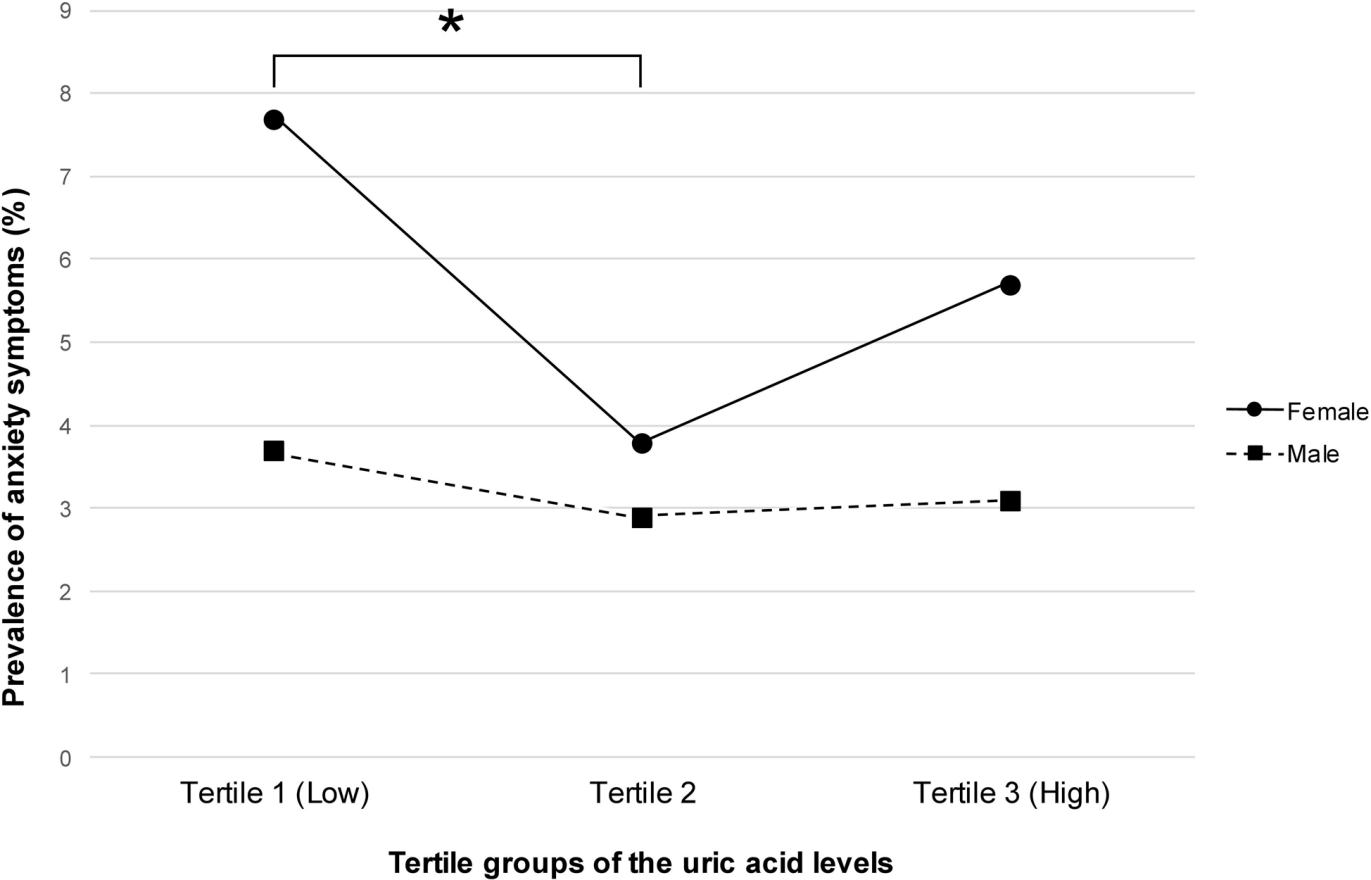
The prevalence of anxiety according to uric acid tertiles in both sexes. * *p* < 0.05

Table 1 shows sociodemographic characteristics of study participants and anxiety symptoms. Among the 5,033 included participants, 72 (3.2%) males and 162 (5.8%) females showed anxiety symptoms (i.e., GAD-7 ≥ 10). The prevalence of anxiety symptoms was not statistically different among UA tertile groups in males. However, the prevalence of anxiety symptoms in females was statistically different among UA tertile groups (*p* = 0.001). In general, for both sexes, participants with anxiety symptoms tended to be unmarried and smokers. Females with anxiety symptoms tended to be 20–39 years old. The levels of UA according to tertiles are shown in Table 2. In all tertiles, it was found that the UA level of females was lower than that of males. The differences in UA levels across each tertile are presented in Fig. 2. To perform a post-hoc test, we conducted a pairwise chi-square analysis of the three tertiles, and a *p*-value lower than 0.05/3 was only present between Tertile 1 (low) and Tertile 2 of the female group (*p* < 0.001).

**Table 1:** Sociodemographic characteristics of study participants and anxiety symptoms (GAD-7 ≥ 10)

	Male				Female		
	Anxiety (*N* = 72)	No anxiety (*N* = 2156)	*p*-value		Anxiety (*N* = 162)	No anxiety (*N* = 2643)	*p*-value
**Serum uric acid levels**			0.666				**0.001**
Tertile 1 (Low)	28 (3.7)	732 (96.3)			73 (7.7)	870 (92.3)	
Tertile 2	21 (2.9)	709 (97.1)			34 (3.8)	867 (96.2)	
Tertile 3 (High)	23 (3.1)	715 (96.9)			55 (5.7)	906 (94.3)	
**Age (years)**			0.054				**< .001**
20–39	21 (3.8)	529 (96.2)			59 (9.3)	579 (90.7)	
40–59	32 (4.0)	762 (96.0)			36 (3.5)	1008 (96.5)	
60 and above	19 (2.2)	865 (97.8)			67 (6.0)	1056 (94.0)	
**Educational attainment**			0.864				0.515
Elementary school and below	12 (4.0)	285 (96.0)			43 (6.8)	594 (93.2)	
Middle school	7 (3.4)	200 (96.6)			17 (5.7)	282 (94.3)	
High school	25 (3.1)	778 (96.9)			52 (6.0)	817 (94.0)	
University or above	28 (3.0)	893 (97.0)			50 (5.0)	950 (95.0)	
**Equalized household income**			0.242				0.066
Quartile 1 (low)	18 (4.9)	347 (95.1)			39 (6.8)	538 (93.2)	
Quartile 2	16 (3.1)	498 (96.9)			38 (5.7)	634 (94.3)	
Quartile 3	20 (3.1)	616 (96.9)			52 (6.9)	701 (93.1)	
Quartile 4 (high)	18 (2.5)	695 (97.5)			33 (4.1)	770 (95.9)	
**Marital status**			**0.039**				**< .001**
Married	41 (2.7)	1490 (97.3)			76 (4.3)	1708 (95.7)	
Not married	31 (4.5)	666 (95.5)			86 (8.4)	935 (91.6)	
**Alcohol use status**			0.537				0.133
No	28 (3.6)	747 (96.4)			93 (5.2)	1681 (94.8)	
Yes	44 (3.0)	1409 (97.0)			69 (6.7)	962 (93.3)	
**Smoking status**			**0.002**				**< .001**
Non-smoker	41 (2.5)	1597 (97.5)			146 (5.4)	2551 (94.6)	
Smoker	31 (5.3)	559 (94.7)			16 (14.8)	92 (85.2)	
**Chronic medical disease**			0.091				0.248
None	38 (3.0)	1233 (97.0)			88 (5.3)	1575 (94.7)	
1 disease	11 (2.3)	463 (97.7)			41 (7.2)	526 (92.8)	
2 or more diseases	23 (4.8)	460 (95.2)			33 (5.7)	542 (94.3)	
**Region**			0.123				0.138
Urban	62 (3.6)	1676 (96.4)			135 (6.1)	2063 (93.9)	
Rural	10 (2.0)	480 (98.0)			27 (4.5)	580 (95.5)	
**BMI**			0.340				0.180
Underweight	3 (4.8)	60 (95.2)			15 (9.7)	139 (90.3)	
Normal weight	14 (2.3)	597 (97.7)			60 (5.1)	1119 (94.9)	
Overweight	24 (4.0)	578 (96.0)			33 (5.7)	545 (94.3)	
Obesity	31 (3.3)	921 (96.7)			54 (6.0)	840 (94.0)	

*GAD-7* Generalized Anxiety Disorder seven-item scale, *BMI* body mass index

**Table 2:** Laboratory ranges for uric acid in all participants by sex and tertile

	Male					Femal**e**				
	Mean	SD	Min		Max	Mean	SD	Min		Max
**Serum uric acid levels**										
Tertile 1 (Low)	4.60	0.68	(1.40	-	5.40)	3.49	0.45	(1.80	-	4.00)
Tertile 2	5.99	0.31	(5.50	-	6.50)	4.44	0.23	(4.10	-	4.80)
Tertile 3 (High)	7.50	0.80	(6.60	-	12.30)	5.67	0.81	(4.90	-	12.60)

*SD* standard deviation, serum uric acid levels in mg/dl

### Sex differences in the association between UA levels and anxiety symptoms

**Fig. 3:**
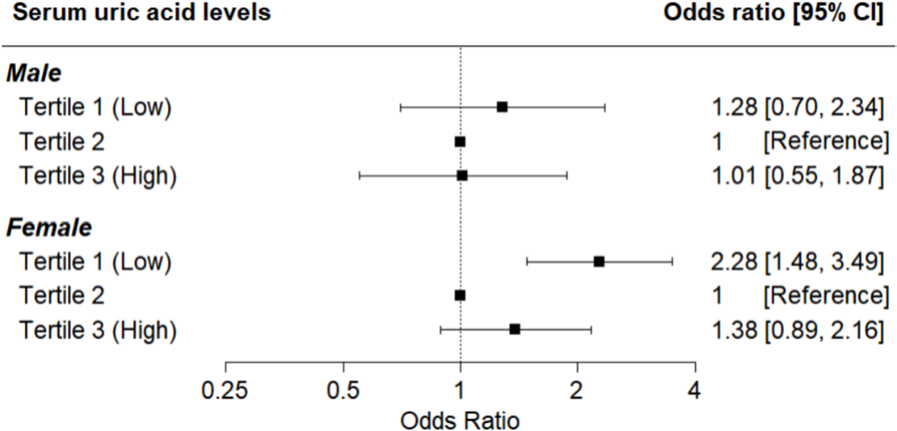
Results of multivariable logistic regression analysis for between uric acid and anxiety disorder (GAD-7 ≥ 10). Results of the multivariable logistic regression analysis for the association between uric acid and anxiety disorder (GAD-7 ≥ 10). Adjusted for educational attainment level, equalized household income, marital status, residential area, alcohol use status, smoking status, number of chronic medical diseases, and body mass index category. GAD-7, Generalized Anxiety Disorder 7-item scale; BMI, body mass index; CI, confidence interval

Figure 3 shows the forest plots of multivariable logistic regression analysis for the association of anxiety symptoms with serum UA levels. In males, there was no statistically significant relationship between anxiety symptoms in the lowest group (Tertile 1) and those in the highest group (Tertile 3) compared to the middle group (Tertile 2) according to serum UA after adjustment for covariates. In contrast, a significant association was observed between anxiety symptoms and serum UA levels in females. Compared to the reference group of serum UA (Tertile 2), the lowest serum UA group (Tertile 1) was approximately 2.28 times more likely to have anxiety symptoms (OR 2.28, 95% CI 1.48–3.49). The confidence intervals for males overlapped substantially across tertiles, contrasting with the distinct associations observed in 2 group of female tertiles.

Table 3 shows the results of multivariable logistic regression analysis for the association between adjusted variables and anxiety symptoms. Age older than 60, high school education level, and high income were found to be associated with a low presence of anxiety symptoms in males. In contrast, smoking, urban residential area, and two or more chronic medical diseases were found to be associated with a high presence of anxiety symptoms in males. Aged younger than 40, unmarried status, smoking, and having one chronic medical disease were associated with an increased prevalence of anxiety symptoms in females.

**Table 3:** Results of multivariable logistic regression analysis between the adjusted variables and anxiety symptoms (GAD-7 ≥ 10)

	Anxiety symptoms (GAD-7 ≥ 10)									
	Male					Female				
	OR	95% CI			*p*-value	OR	95% CI			*p*-value
**Age (years)**										
20–39	1.00					1.00				
40–59	0.88	0.45	-	1.71	0.696	**0.35**	**0.22**	**-**	**0.58**	** < .001**
60 and above	**0.24**	**0.09**	**-**	**0.63**	**0.004**	**0.44**	**0.23**	**-**	**0.83**	**0.011**
**Educational attainment**										
Elementary school and below	1.00					1.00				
Middle school	0.64	0.23	-	1.76	0.390	0.85	0.45	-	1.58	0.599
High school	**0.41**	**0.17**	**-**	**0.98**	**0.045**	0.84	0.47	-	1.51	0.565
University or above	0.44	0.17	-	1.10	0.079	0.70	0.37	-	1.33	0.270
**Equalized household income**										
Quartile 1 (low)	1.00					1.00				
Quartile 2	0.54	0.26	-	1.13	0.100	0.98	0.59	-	1.62	0.933
Quartile 3	0.56	0.27	-	1.18	0.125	1.23	0.74	-	2.06	0.429
Quartile 4 (high)	**0.42**	**0.19**	**-**	**0.94**	**0.035**	0.77	0.44	-	1.38	0.383
**Marital status**										
Married	1.00					1.00				
Not married	1.26	0.72	-	2.21	0.424	**1.53**	**1.07**	-	**2.18**	**0.019**
**Region**										
Urban	1.00					1.00				
Rural	**0.48**	**0.24**	**-**	**0.98**	**0.044**	0.69	0.44	-	1.07	0.096
**Alcohol use status**										
No	1.00					1.00				
Yes	0.70	0.42	-	1.17	0.178	1.31	0.91	-	1.88	0.142
**Smoking status**										
Non-smoker	1.00					1.00				
Smoker	**2.02**	**1.22**	**-**	**3.34**	**0.006**	**2.69**	**1.49**	**-**	**4.87**	**0.001**
**Chronic disease**										
None	1.00					1.00				
1 disease	1.15	0.54	-	2.44	0.723	**2.02**	**1.24**	**-**	**3.29**	**0.005**
2 or more diseases	**2.28**	**1.19**	**-**	**4.36**	**0.013**	1.51	0.87	-	2.62	0.142
**BMI**										
Underweight	2.01	0.54	-	7.46	0.296	1.61	0.87	-	2.98	0.128
Normal weight	1.00					1.00				
Overweight	1.90	0.96	-	3.76	0.066	1.16	0.73	-	1.82	0.538
Obesity	1.43	0.74	-	2.77	0.297	1.25	0.83	-	1.89	0.292

Adjusted for the covariates listed above as well as serum uric acid level

*GAD-7* Generalized Anxiety Disorder seven-item scale, *BMI* body mass index, *OR* odds ratio, *CI* confidence interval

### Association between UA and anxiety symptoms stratified by sex and age group

The results of subgroup analysis showing the association between UA and anxiety symptoms stratified by sex and age group are presented in Supplementary Table S1, Appendix file. The participants were divided into four groups by sex and age. There was no significant association observed between anxiety symptoms and serum UA levels for the older and younger male groups. In females, the older female group with the lowest UA levels (Tertile 1) was 2.86 times more likely to have anxiety symptoms than that with middle UA levels (Tertile 2; OR 2.86, 95% CI 1.30–6.32, *p* = 0.009). The younger female group (age < 65) with the lowest UA level (Tertile1) was 2.03 times more likely to have anxiety symptoms than that with middle UA levels (Tertile 2; OR 2.03, 95% CI 1.22–3.38, *p* = 0.006).

### Sex and age differences in the association between UA levels and anxiety symptoms using a different GAD-7 cutoff score

Supplementary Figure S1, Appendix file and Supplementary Table S2, Appendix file show the results of multivariable logistic regression analysis for the association of anxiety symptoms based on a GAD-7 score of 5 or more and serum UA levels. In males, there was no statistically significant relationship between anxiety symptoms and serum UA after adjustment for covariates in any groups (Tertile 1 and 3) compared to the middle group (Tertile 2). In contrast, a significant association was observed between anxiety symptoms and serum UA levels in females. Compared to the reference group for serum UA (Tertile 2), the lowest serum UA group (Tertile 1) was 1.68 times more likely to have anxiety symptoms (OR 1.68, 95% CI 1.30–2.1), and the highest serum UA group (tertile 3) was 1.35 times more likely to have anxiety symptoms (OR 1.35, 95% CI 1.04–1.74). The confidence intervals for males extended over each other tertiles, in contrast to the clear separations identified in females.

The results of subgroup analysis showing the association between UA and anxiety symptoms stratified by sex and age group (i.e., GAD-7 ≥ 5) are presented in Supplementary Table S3, Appendix file. There was no significant association observed between anxiety symptoms and serum UA levels for all male groups. In the older female group, participants with the lowest UA levels (Tertile 1) were 1.99 times as likely to have anxiety symptoms as those with middle UA levels (Tertile 2; OR 1.99, 95% CI 1.18–3.37, *p* = 0.010). The younger female group (age < 65) with the lowest UA level (Tertile 1) was 1.53 times more likely to have anxiety symptoms than that with middle UA levels (Tertile 2; OR 1.53, 95% CI 1.15–2.04, *p* = 0.003), and the group with the highest UA levels (Tertile 3) was also 1.35 times more likely to have anxiety symptoms than that with middle UA levels (Tertile 2; OR 1.35, 95% CI 1.01–1.81, *p* = 0.042).

### Verification of appropriateness of multiple logistic regression analysis

For all datasets and independent variables, all VIF values were below the commonly accepted threshold of 10, indicating no significant multicollinearity that would undermine the model's stability. None of the observations had Cook’s Distance values greater than 1 or DFBETA values beyond the critical threshold (± 1), confirming that no single data point was unduly influencing the regression results. The *p*-value from Hosmer–Lemeshow test was greater than 0.05, indicating that the model’s predicted probabilities aligned well with the observed outcomes, suggesting an adequate fit for the data. These steps collectively confirm that the use of multivariable logistic regression was appropriate for the analysis, and the model's assumptions were adequately met.

## Discussion

This study shows that females with lower UA levels (Tertile 1) were more likely to exhibit anxiety symptoms than females with middle UA levels (Tertile 2), but females with higher UA levels (Tertile 3) were not statistically different from females with middle UA levels (Tertile 2). In males, no significant difference in terms of the prevalence of anxiety symptoms was found between tertiles based on serum UA levels.

The fact that plasma UA levels are lower in current but not remitted anxiety disorders was revealed in a previous study [17]. That study differed in that male participants did not show a difference in the presence of anxiety symptoms depending on serum UA levels, and not all female groups showed an inverse association with plasma UA levels and the presence of anxiety symptoms. The difference in the association of UA and anxiety symptoms by sex was similarly shown in the same KNHANES-based study, which indicated that UA is associated with depression in older females but not in males [26]. It is well known that males generally have higher serum UA levels than females [27]. This difference is partly because of sex hormones. Estrogen increases UA excretion in urine [28], leading to generally lower levels in females. Given that females have lower UA levels and less intrinsic reactive oxygen species (ROS) production, it is possible that in females, the protective effects of UA become more pronounced. In other words, with males having generally higher UA levels, a small decrease might not produce as evident a clinical effect as it would in females. The sex-specific differences in hormones, metabolism, and other physiological processes might also influence the vulnerability of females with low UA to developing anxiety disorders.

Recent data have demonstrated that there is a link between oxidative stress and high-anxiety-related behavior, and some of these studies suggest that oxidative stress causes anxiety-related behaviors [13]. Blood levels of specific antioxidants appear in general to be lower in the presence of anxiety, suggesting depletion of non-enzymatic antioxidant defenses [14]. A meta-analysis study showed that antioxidant supplementation elicited significant improvement in anxiety, affirming the therapeutic potential of antioxidant supplements [29]. Recent research has discovered potential antioxidative properties of UA, suggesting that UA may play a protective role against oxidative stress [7]. Plasma levels of UA show a strong correlation with UA levels in cerebrospinal fluid [9], evidencing the antioxidant effect of UA in the CNS. People with reduced UA levels, suggesting weakened antioxidant protection, might be more prone to oxidative stress in the brain, which places them at particular risk because of high oxygen use. These conditions can lead to anxiety-related behaviors, explaining why the anxiety symptoms of the low UA-level group (Tertile 1) were observed significantly more frequently than those of the middle UA-level group (Tertile 2).

The results of multivariate logistic regression analysis of the association of anxiety symptoms, which was considered with a GAD-7 score of 5 or greater, with serum UA levels shows a U-shaped relationship between UA levels and mild anxiety symptoms in females. An observational study indicates a U-shaped association between UA and brain diseases [30], which supports this result. High levels of UA (hyperuricemia) can be associated with gout, kidney stones, and other health problems. The pain, discomfort, and stress of these conditions might exacerbate or even trigger anxiety disorders. Further research should more thoroughly consider hyperuricemia-related diseases that could affect anxiety symptoms in order to support this theory. Estrogen influences UA excretion, and drastic fluctuations in estrogen levels, as seen during menopause and in other hormonal imbalances, might affect UA levels and play a role in mental health. UA itself acts as an antioxidant in the extracellular environment but can also act as a pro-oxidant, mainly in the intracellular environment [31]. This pro-oxidant effect might have caused anxiety symptoms in high serum UA-level group. Future research should explore the distinct biological mechanisms, such as oxidative stress, inflammation, or hormonal regulation, that may underlie this bidirectional association. These findings also underscore the need for longitudinal studies to explore possible non-linear association of UA with anxiety symptoms and determine whether interventions targeting UA levels could mitigate anxiety symptoms, particularly considering that the association may differ by sex.

However, the U-shaped relationship between UA levels and anxiety symptoms in females was not shown with a higher GAD-7 cutoff score of 10. The pro-oxidant effect of UA might not be effective enough to cause moderate anxiety symptoms at significant levels, instead only causing mild anxiety symptoms. The underlying biological and psychological factors contributing to mild anxiety might differ from those contributing to more severe anxiety. At higher anxiety levels, other biochemical factors and structural or functional brain changes might play a more dominant role in anxiety, overshadowing the influence of UA. For example, neurotransmitter imbalances may become more pronounced at severe anxiety levels [32], making the impact of UA less discernible. People with more severe anxiety may have developed certain adaptive or compensatory mechanisms over time. These mechanisms could potentially mask or override the effects of high UA on anxiety. Those with higher GAD-7 scores may be more likely to be on medications or undergoing other therapeutic interventions that could interact with UA metabolism or its effects on the body [33], thus confounding the relationship at higher anxiety levels. Additionally, severe anxiety symptoms may be influenced by chronic stress, which can independently alter UA levels through its effects on cortisol and systemic inflammation [34, 35]. Socioeconomic or environmental stressors, such as financial instability or traumatic life events, may also disproportionately affect individuals with severe anxiety, further complicating the observed relationship.

The associations of covariates and anxiety symptoms are also apparent. Aged younger than 40, unmarried status, smoking status, and having one chronic medical disease morbidity were associated with an increased prevalence of anxiety symptoms in females. Younger adults often grapple with significant life transitions, which can induce heightened anxiety symptoms. Married people exhibit greater emotional and psychological well-being compared to their single, divorced, or cohabiting counterparts. [36, 37]. Previous studies have suggested that individuals with increased anxiety are more likely to smoke [38, 39]; thus, smoking status being associated with an increased prevalence of anxiety symptoms in females is predictable. Having a chronic disease is associated with lower levels of antioxidant substances [40]. Chronic diseases also create physical stresses and worry regarding health, prognosis, and impact on daily life. These factors can lead to increased anxiety symptoms.

As with females, smoking and two or more chronic medical diseases were found to be associated with a high presence of anxiety symptom, and being older than 60 was associated with a low presence of anxiety symptoms in males. In contrast, high school education and high income were associated with a low presence of anxiety symptoms, and an urban residential area was associated with a high presence of anxiety symptoms in males. A high level of education and high income are commonly recognized as reflecting social success in Korean society [41]; thus, feelings of achievement and respect from others may lead to greater emotional and psychological well-being and, thus, fewer anxiety symptoms. Moreover, for anxiety disorders, the pooled urban prevalence rate was higher in urban areas compared to rural areas [42], which is similarly shown in this study.

In addition, 3.2% of males and 5.8% of females showed anxiety symptoms (i.e., GAD-7 ≥ 10) in this study. In prior study, the 12-month prevalence rate of anxiety disorder was 1.6% in males and 4.7% in females among general Korean population [43]. This study shows a slightly lower prevalence rate than our study, and the difference in prevalence rate seems to be because of the difference of diagnostic tools. The prior study used the Korean version of the Composite International Diagnostic Interview (CIDI) 2.1. performed by trained interviewers [44]. The tool we used is GAD-7, a self-screening tool to diagnose generalized anxiety disorder. Although GAD-7 is a reliable anxiety screening tool, the prevalence rate will be slightly higher than the diagnostic tool performed by trained interviewers.

This study has several strengths. First, it revealed a correlation between serum UA levels and the occurrence of anxiety symptoms in female adults using comprehensive and consistent nationwide data from a large sample. Moreover, a GAD-7 cutoff score of 5 showed a U-shaped relationship between UA levels and mild anxiety symptoms in females via subgroup analysis, which deviated from the results of previous studies. These findings highlight the importance of considering UA levels as part of a broader investigation into anxiety's biological underpinnings. However, this study also has limitations. It is based on cross-sectional data analysis, providing only a snapshot of data at a single point in time. This is linked with an inability to draw causal conclusions. These findings should be viewed with caution, as they do not consider changes over time or potential causal mechanisms. The concentration of a single marker such as UA in this context, at one specific moment, may not capture the overall functioning of the redox-homeostasis system. Furthermore, factors such as diet, supplements, heart medications such as blood pressure reducers and diuretics, and urate-lowering agents, which might influence serum UA levels, were not fully adjusted. The factors that affect serum UA levels like daily meat consumption [45], fructose intake [46], serum estrogen levels [28], physical activity levels [47] were not included in this study. Considering these factors in future analyses is necessary, as their inclusion could influence the observed association between serum UA and anxiety. The observed association may be shaped by a complex network of interconnected factors rather than existing independently. There may be other potential unadjusted confounding factors influencing the relationship, therefore interpretation of results should be done in caution.

## Conclusion

In conclusion, our study indicates that low levels of serum UA are associated with a higher prevalence of anxiety symptoms in females only, suggesting the involvement of oxidative stress in anxiety disorder. However, this association is still in its early stages and should be seen as a starting point for further research rather than a decisive conclusion. Future research is necessary to investigate the relationship between UA and anxiety disorder using longitudinal studies, randomized controlled trials, or other prospective designs to clarify causality. Additionally, research needs to explore interrelated factors to provide clearer insights into the fundamental interactions of anxiety.

## Supplementary Information

Supplementary Material 1. Results of supplementary analysis. Results of the multivariable logistic regression analysis for the association between uric acid and anxiety symptoms and subgroup analysis for the association between uric acid and anxiety symptoms stratified by sex and age group in different criteria of GAD-7 score.

## Data Availability

The datasets used during the current study are available from the homepage of the Korea Disease Control and Prevention (https://knhanes.kdca.go.kr/knhanes/sub03/sub03_01.do).

## Abbreviations

GAD-7: Generalized Anxiety Disorder 7-item scale
OR: Odds ratio
CI: Confidence interval
GAD: Generalized anxiety disorder
DALY: Disability-adjusted life years lost
UA: Uric acid
CNS: Central nervous system
MDD: Major depressive disorder
KNHANES: Korea National Health and Nutrition Examination Survey
ROC: Receiver operating characteristic
ROS: Reactive oxygen species
CIDI: Composite International Diagnostic Interview
VIF: Variance Inflation Factor
DFBETA: Difference in Betas
