# Supplementary material for: Sex-based disparities in the association between uric acid levels and anxiety: a cross-sectional analysis of nationwide data in Korea
Source: BMC Psychiatry. 2025 Jan 23;25:69. doi: 10.1186/s12888-025-06474-3 (PMC11760062; doi:10.1186/s12888-025-06474-3)
Supplement: Supplementary file 1 — Supplementary Material 1. Results of supplementary analysis. Results of the multivariable logistic regression analysis for the association between uric acid and anxiety symptoms and subgroup analysis for the association between uric acid and anxiety symptoms stratified by sex and age group in different criteria of GAD-7 score. [file 12888_2025_6474_MOESM1_ESM.docx]

**Appendix**

**Sex-Based Disparities in the Association Between Uric Acid Levels and Anxiety: A Cross-Sectional Analysis of Nationwide Data in Korea**

**Table of Contents**

[**Supplementary Figure S1. Results of multivariable logistic regression analysis between uric acid and anxiety symptoms (GAD-7 ≥5)** 2](#_Toc146260946)

[**Supplementary Table S1. Results of the subgroup analysis between uric acid and anxiety symptoms (GAD-7 ≥ 10)** 3](#_Toc146260947)

[**Supplementary Table S2. Results of multivariable logistic regression analysis between uric acid and anxiety symptoms (GAD-7 ≥5)** 4](#_Toc146260948)

[**Supplementary Table S3. Results of the subgroup analysis between uric acid and anxiety symptoms (GAD-7 ≥ 5)** 5](#_Toc146260949)

# **Supplementary Figure S1. Results of multivariable logistic regression analysis between uric acid and anxiety symptoms (GAD-7 ≥5)**


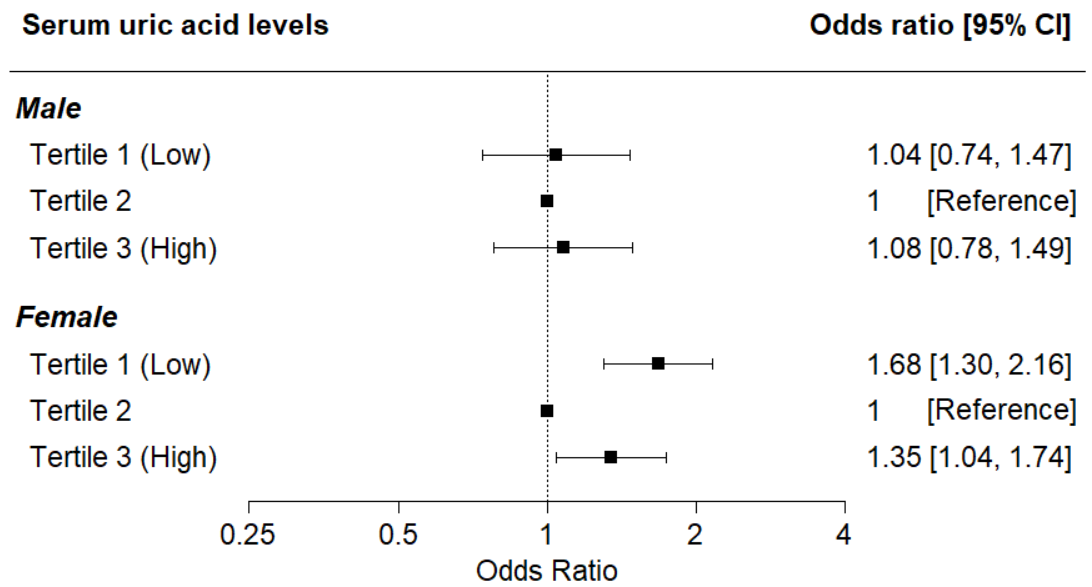


Results of the multivariable logistic regression analysis for the association between uric acid and anxiety symptoms (GAD-7 ≥5). Adjusted for educational attainment level, equalized household income, marital status, residential area, alcohol use status, smoking status, number of chronic medical diseases, and body mass index category; GAD-7, Generalized Anxiety Disorder seven-item scale; BMI, body mass index; CI, confidence interval

| **Supplementary Table S1. Results of the subgroup analysis between uric acid and anxiety symptoms (GAD-7 ≥ 10)** | | | | | | | | | | |
| --- | --- | --- | --- | --- | --- | --- | --- | --- | --- | --- |
|  | **Anxiety symptoms (GAD-7 ≥10)** | | | | | | | | | |
|  | **Male** | | | | | **Female** | | | | |
|  | **OR** | **95% CI** | | | ***p*-value** | **OR** | **95% CI** | | | ***p*-value** |
| **Younger participants**  **(Age <65)** |  |  |  |  |  |  |  |  |  |  |
| **Serum uric acid levels** |  |  |  |  |  |  |  |  |  |  |
| Tertile 1 (Low) | 0.94 | 0.49 | - | 1.81 | 0.858 | **2.03** | **1.22** | **-** | **3.38** | **0.006** |
| Tertile 2 | 1.00 |  |  |  |  | 1.00 |  |  |  |  |
| Tertile 3 (High) | 0.92 | 0.47 | - | 1.79 | 0.800 | 1.42 | 0.84 | - | 2.41 | 0.195 |
| **Older participants**  **(Age ≥65)** |  |  |  |  |  |  |  |  |  |  |
| **Serum uric acid levels** |  |  |  |  |  |  |  |  |  |  |
| Tertile 1 (Low) | 7.21 | 0.82 | - | 63.25 | 0.075 | **2.86** | **1.30** | **-** | **6.32** | **0.009** |
| Tertile 2 | 1.00 |  |  |  |  | 1.00 |  |  |  |  |
| Tertile 3 (High) | 6.10 | 0.68 | - | 54.93 | 0.107 | 1.45 | 0.60 | - | 3.47 | 0.410 |
| Results of the subgroup analysis for the association between uric acid and anxiety symptoms stratified by sex and age group (GAD-7 ≥ 10). Adjusted for educational attainment level, equalized household income, marital status, residential area, alcohol use status, smoking status, number of chronic medical diseases, and body mass index category;  GAD-7, Generalized Anxiety Disorder seven-item scale; BMI, body mass index; OR, odds ratio; CI, confidence interval | | | | | | | | | | |

| **Supplementary Table S2. Results of multivariable logistic regression analysis between uric acid and anxiety symptoms (GAD-7 ≥5)** | | | | | | | | | | |
| --- | --- | --- | --- | --- | --- | --- | --- | --- | --- | --- |
|  | **Anxiety symptoms (GAD-7 ≥ 5)** | | | | | | | | | |
|  | **Male** | | | | | **Female** | | | | |
|  | **OR** | **95% CI** | | | ***p*-value** | **OR** | **95% CI** | | | ***p*-value** |
| **Age (years)** |  |  |  |  |  |  |  |  |  |  |
| 20-39 | 1.00 |  |  |  |  | 1.00 |  |  |  |  |
| 40-59 | **0.59** | **0.41** | **-** | **0.84** | **0.004** | **0.50** | **0.38** | **-** | **0.66** | **<.001** |
| 60 and above | **0.32** | **0.19** | **-** | **0.53** | **<.001** | **0.33** | **0.22** | **-** | **0.49** | **<.001** |
| **Educational attainment** |  |  |  |  |  |  |  |  |  |  |
| Elementary school and below | 1.00 |  |  |  |  | 1.00 |  |  |  |  |
| Middle school | 0.94 | 0.51 | - | 1.73 | 0.850 | 1.00 | 0.67 | - | 1.50 | 0.998 |
| High school | 0.59 | 0.35 | - | 1.00 | 0.049 | 1.00 | 0.69 | - | 1.46 | 0.994 |
| University or above | 0.81 | 0.47 | - | 1.40 | 0.452 | 0.93 | 0.62 | - | 1.40 | 0.725 |
| **Equalized household income** |  |  |  |  |  |  |  |  |  |  |
| Quartile 1 (low) | 1.00 |  |  |  |  | 1.00 |  |  |  |  |
| Quartile 2 | 1.05 | 0.67 | - | 1.65 | 0.821 | 0.97 | 0.71 | - | 1.34 | 0.857 |
| Quartile 3 | 0.96 | 0.61 | - | 1.51 | 0.868 | 0.74 | 0.53 | - | 1.04 | 0.084 |
| Quartile 4 (high) | 0.73 | 0.46 | - | 1.17 | 0.190 | 0.81 | 0.57 | - | 1.14 | 0.223 |
| **Marital status** |  |  |  |  |  |  |  |  |  |  |
| Married | 1.00 |  |  |  |  | 1.00 |  |  |  |  |
| Not married | **1.41** | **1.03** | **-** | **1.95** | **0.034** | **1.53** | **1.23** | **-** | **1.91** | **<.001** |
| **Region** |  |  |  |  |  |  |  |  |  |  |
| Urban | 1.00 |  |  |  |  | 1.00 |  |  |  |  |
| Rural | **0.67** | **0.46** | **-** | **0.96** | **0.031** | 0.82 | 0.63 | - | 1.06 | 0.129 |
| **Alcohol use status** |  |  |  |  |  |  |  |  |  |  |
| No | 1.00 |  |  |  |  | 1.00 |  |  |  |  |
| Yes | 1.17 | 0.87 | - | 1.58 | 0.292 | **1.26** | **1.01** | **-** | **1.56** | **0.040** |
| **Smoking status** |  |  |  |  |  |  |  |  |  |  |
| Non-smoker | 1.00 |  |  |  |  | 1.00 |  |  |  |  |
| Smoker | 1.27 | 0.95 | - | 1.71 | 0.112 | **1.67** | **1.07** | **-** | **2.59** | **0.024** |
| **Chronic disease** |  |  |  |  |  |  |  |  |  |  |
| None | 1.00 |  |  |  |  | 1.00 |  |  |  |  |
| 1 disease | 0.95 | 0.63 | - | 1.45 | 0.823 | **1.48** | **1.10** | **-** | **2.00** | **0.009** |
| 2 or more diseases | **1.65** | **1.12** | **-** | **2.43** | **0.011** | 1.32 | 0.95 | - | 1.85 | 0.097 |
| **BMI** |  |  |  |  |  |  |  |  |  |  |
| Underweight | 1.01 | 0.43 | - | 2.36 | 0.988 | 1.16 | 0.77 | - | 1.75 | 0.486 |
| Normal weight | 1.00 |  |  |  |  | 1.00 |  |  |  |  |
| Overweight | 1.37 | 0.95 | - | 1.96 | 0.092 | 1.01 | 0.76 | - | 1.33 | 0.969 |
| Obesity | 1.06 | 0.75 | - | 1.51 | 0.735 | 1.09 | 0.85 | - | 1.39 | 0.518 |
| Results of the multivariable logistic regression analysis for the association between uric acid and anxiety symptoms (GAD-7 ≥5). Adjusted for covariates listed above and serum uric acid levels; GAD-7, Generalized Anxiety Disorder seven-item scale; BMI, body mass index; OR, odds ratio; CI, confidence interval | | | | | | | | | | |

| **Supplementary Table S3. Results of the subgroup analysis between uric acid and anxiety symptoms (GAD-7 ≥ 5)** | | | | | | | | | | |
| --- | --- | --- | --- | --- | --- | --- | --- | --- | --- | --- |
|  | **Anxiety symptoms (GAD-7 ≥ 5)** | | | | | | | | | |
|  | **Male** | | | | | **Female** | | | | |
|  | **OR** | **95% CI** | | | ***p*-value** | **OR** | **95% CI** | | | ***p*-value** |
| **Younger participants (Age < 65)** |  |  |  |  |  |  |  |  |  |  |
| **Serum uric acid levels** |  |  |  |  |  |  |  |  |  |  |
| Tertile 1 (Low) | 0.96 | 0.67 | - | 1.37 | 0.806 | **1.53** | **1.15** | **-** | **2.04** | **0.003** |
| Tertile 2 | 1.00 |  |  |  |  | 1.00 |  |  |  |  |
| Tertile 3 (High) | 1.06 | 0.74 | - | 1.52 | 0.763 | **1.35** | **1.01** | **-** | **1.81** | **0.042** |
| **Older participants**  **(Age ≥ 65)** |  |  |  |  |  |  |  |  |  |  |
| **Serum uric acid levels** |  |  |  |  |  |  |  |  |  |  |
| Tertile 1 (Low) | 0.96 | 0.44 | - | 2.10 | 0.920 | **1.99** | **1.18** | **-** | **3.37** | **0.010** |
| Tertile 2 | 1.00 |  |  |  |  | 1.00 |  |  |  |  |
| Tertile 3 (High) | 0.80 | 0.36 | - | 1.80 | 0.595 | 1.44 | 0.83 | - | 2.49 | 0.198 |
| Results of the subgroup analysis for the association between uric acid and anxiety symptoms stratified by sex and age group (GAD-7 ≥ 5). Adjusted for educational attainment level, equalized household income, marital status, residential area, alcohol use status, smoking status, number of chronic medical diseases, and body mass index category;  GAD-7, Generalized Anxiety Disorder seven-item scale; BMI, body mass index; OR, odds ratio; CI, confidence interval | | | | | | | | | | |
